# The UBA-UIM Domains of the USP25 Regulate the Enzyme Ubiquitination State and Modulate Substrate Recognition

**DOI:** 10.1371/journal.pone.0005571

**Published:** 2009-05-15

**Authors:** Amanda Denuc, Anna Bosch-Comas, Roser Gonzàlez-Duarte, Gemma Marfany

**Affiliations:** 1 Departament de Genètica, Facultat de Biologia, Universitat de Barcelona, Barcelona, Spain; 2 Institut de Biomedicina de la Universitat de Barcelona (IBUB), Barcelona, Spain; 3 Centre for Biomedical Research on Rare Diseases (CIBERER), Instituto de Salud Carlos III, Barcelona, Spain; University of Munich and Center of Integrated Protein Science, Germany

## Abstract

USP25m is the muscle isoform of the deubiquitinating (DUB) enzyme USP25. Similarly to most DUBs, data on USP25 regulation and substrate recognition is scarce. *In silico* analysis predicted three ubiquitin binding domains (UBDs) at the N-terminus: one ubiquitin-associated domain (UBA) and two ubiquitin-interacting motifs (UIMs), whereas no clear structural homology at the extended C-terminal region outside the catalytic domains were detected. In order to asses the contribution of the UBDs and the C-terminus to the regulation of USP25m catalytic activity, ubiquitination state and substrate interaction, serial and combinatorial deletions were generated. Our results showed that USP25m catalytic activity did not strictly depend on the UBDs, but required a coiled-coil stretch between amino acids 679 to 769. USP25 oligomerized but this interaction did not require either the UBDs or the C-terminus. Besides, USP25 was monoubiquitinated and able to autodeubiquitinate in a possible loop of autoregulation. UBDs favored the monoubiquitination of USP25m at the preferential site lysine 99 (K99). This residue had been previously shown to be a target for SUMO and this modification inhibited USP25 activity. We showed that mutation of K99 clearly diminished USP25-dependent rescue of the specific substrate MyBPC1 from proteasome degradation, thereby supporting a new mechanistic model, in which USP25m is regulated through alternative conjugation of ubiquitin (activating) or SUMO (inhibiting) to the same lysine residue (K99), which may promote the interaction with distinct intramolecular regulatory domains.

## Introduction

Ubiquitin (Ub) modifies protein architecture when covalently attached to its substrates. Besides being the main tag for sending misfolded proteins to the proteasome, Ub also plays a relevant role in protein-protein interaction and modulation of catalytic activity or protein fate [1]–[3]. The intrincate Ub-signalling networks require a tight regulation of both conjugation and deconjugation processes, and the final fate of the modified protein depends on several factors, including the ubiquitin chain length and the configuration of Ub-Ub linkages within the poly-Ub chain [4], [5]. In particular, monoubiquitination is not related to proteasome targeting but to modification of enzymatic activity and subcellular localization [6], [7]. On the other hand, ubiquitin-like molecules (Ubls), such as SUMO, are also covalently bound to their substrates, and thus are conjugated, deconjugated and recognized by specific enzymes and their targets [8], [9].

Although many studies have investigated the activation of Ub and its transfer to substrates [10], the biochemical mechanisms downstream of ubiquitination are not completely understood. It is known that the subsequent events are mediated by ubiquitin receptors, which interact with monoubiquitin and/or polyubiquitin chains through small (20–150 amino acids) Ub-binding domains (UBDs) [11], [12]. At least fifteen classes of UBDs have been annotated [13] and this profusion of motifs has launched the study of Ub signalling by: i) providing clues on the roles and modes of action of ubiquitinated substrates, and ii) showing that UBD-containing proteins interact either with Ub or with a ubiquitinated protein. UBD-Ub interactions are usually weak and generate a dynamic protein network that is rapidly assembled and disassembled, thus hindering their study. Moreover, UBDs can modulate the activity of the host protein, as intramolecular interactions between a UBD and a Ub moiety covalently attached to another region of the same protein lead to structural changes that alter the enzymatic activity [11], [12].

UBDs are found not only in proteins that interact with ubiquitinated substrates, but also in ubiquitinating or deubiquitinating enzymes. The deubiquitinating enzymes (DUBs) hydrolyze the Ub moieties conjugated to substrates and thus, process newly synthesized Ub, recycle Ub, or edit polyUb chains [14], [15]. Ubiquitination, like phosphorylation, is reversible [16] and, therefore, DUBs can affect the stability and fate of Ub-conjugated proteins, and also allow a tight control of Ub-induced switches. It is assumed that the presence of UBDs in DUBs favor the specific recognition of the ubiquitin modifications, whereas the N- and C-terminal long extensions flanking the DUB-conserved catalytic core may be involved in substrate recognition irrespective of their ubiquitination state.

Data on the substrate specificity and physiological function of most DUBs, including USP25, are still scanty. *USP25* encodes three different protein isoforms produced by alternative splicing: two of them are expressed ubiquitously, while the longest (USP25m) is restricted to muscle tissues [17] and is upregulated during myogenesis. Among several sarcomeric substrates, USP25m was reported to specifically interact and rescue MyBPC1 (Myosin Binding Protein C1) from proteasome degradation, thereby raising its cellular half-life [18].

We aimed to identify structural domains relevant for USP25m regulation. By *in silico* analysis we identified three potential UBD signatures in the N-terminal region of USP25m. Here, we characterized USP25 by assessing the contribution of these UBDs, as well as the long C-terminal region of USP25, to the catalytic activity. Our results showed that USP25m was monoubiquitinated in cultured cells, and that the UBDs modulated this modification. The preferential site for monoubiquitination is lysine 99 (K99), a residue that has been recently reported to be also the target of sumoylation [19], [20]. According to our results, mutation of the K99 residue diminishes the rescue of the specific substrate MyBPC1 from proteasome degradation. In view of these results and those of other authors [19], we propose a novel mechanistic model for USP25m regulation in which the same lysine residue can be either ubiquitinated or sumoylated, and these mutually exclusive modifications have opposite effects on the enzyme activity. This regulatory model bridges the Ub and SUMO pathways and may be extrapolated to other ubiquitin-specific proteases.

## Results

### Mutation of the Cys178 catalytic residue abrogates USP25m deubiquitinating activity

**Figure 1:**
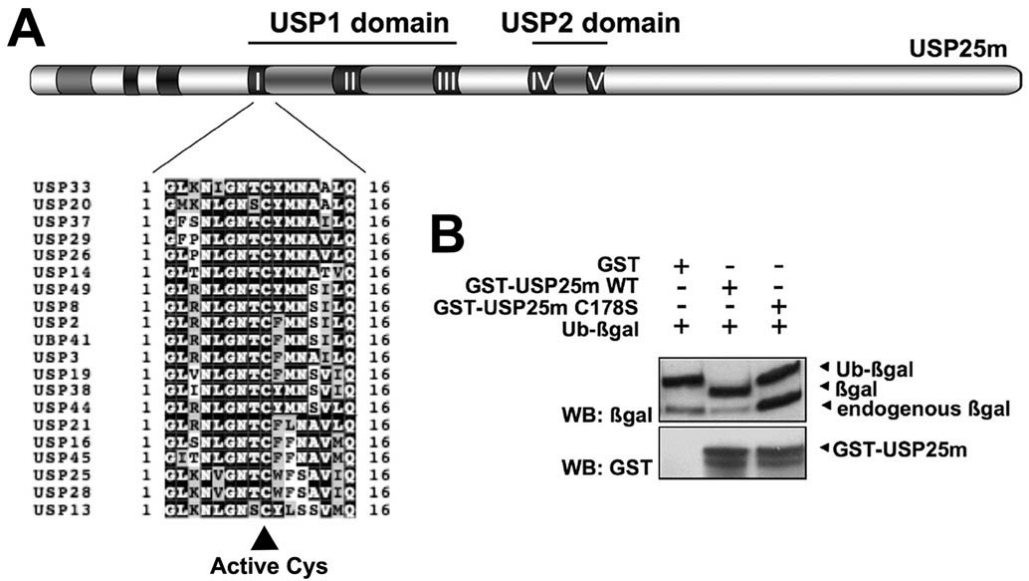
USP25 domain dissection and their contribution to the catalytic activity. A. Sequence homologies revealed five highly conserved USP motifs (I to V) in two domains (USP1 and USP2) that catalogue USP25m as a deubiquitinating enzyme. Cys-178 is the putative active site of the enzyme, since it is conserved in all analyzed members of the family. B. Deubiquitinating activity assays in *E.coli* cells co-transformed with the recombinant substrate Ub-βgalactosidase and either wild type (WT) USP25m or the C178S mutant confirmed that Cys 178 is the active site of USP25m. βgal immunodetectection shows a lower band using WT USP25m, indicating hydrolysis of Ub from βgal, while the mutated form is catalytically inactive and displays the band of the uncleaved fusion Ub-βgal. Note that the endogenous β-galactosidase is of lower molecular weight.

USP25m sequence (1125 aa) alignments revealed five highly conserved distinct motifs (I to V), embedded in two domains (USP1 and USP2) characteristic of the ubiquitin-specific protease family (UBPs, USPs in humans) [17]. The conserved catalytic triad (Cys, Asp and His), in which Cys-178 was the presumed key residue for DUB activity, was located in the motifs I, II and IV, respectively (Figure 1A). Evidence of Cys-178 direct role in USP25m DUB activity was obtained by site-directed mutagenesis to Ser (C178S mutant). A deubiquitinating activity assay for USP25m was used to verify this hypothesis. USP25m and Ub-β-Galactosidase co-transformation in BL21 cells rapidly induced the proteolysis of the fusion between Ub and β-Gal (Figure 1B). This proteolityc activity was not observed with the C178S mutant, thus showing that Cys-178 is essential for the deubiquitinating activity of USP25m.

### The deubiquitinating activity of USP25m depends on the presence of a long coiled-coil stretch, but does not require the N-terminus Ubiquitin Binding Domains

**Figure 2:**
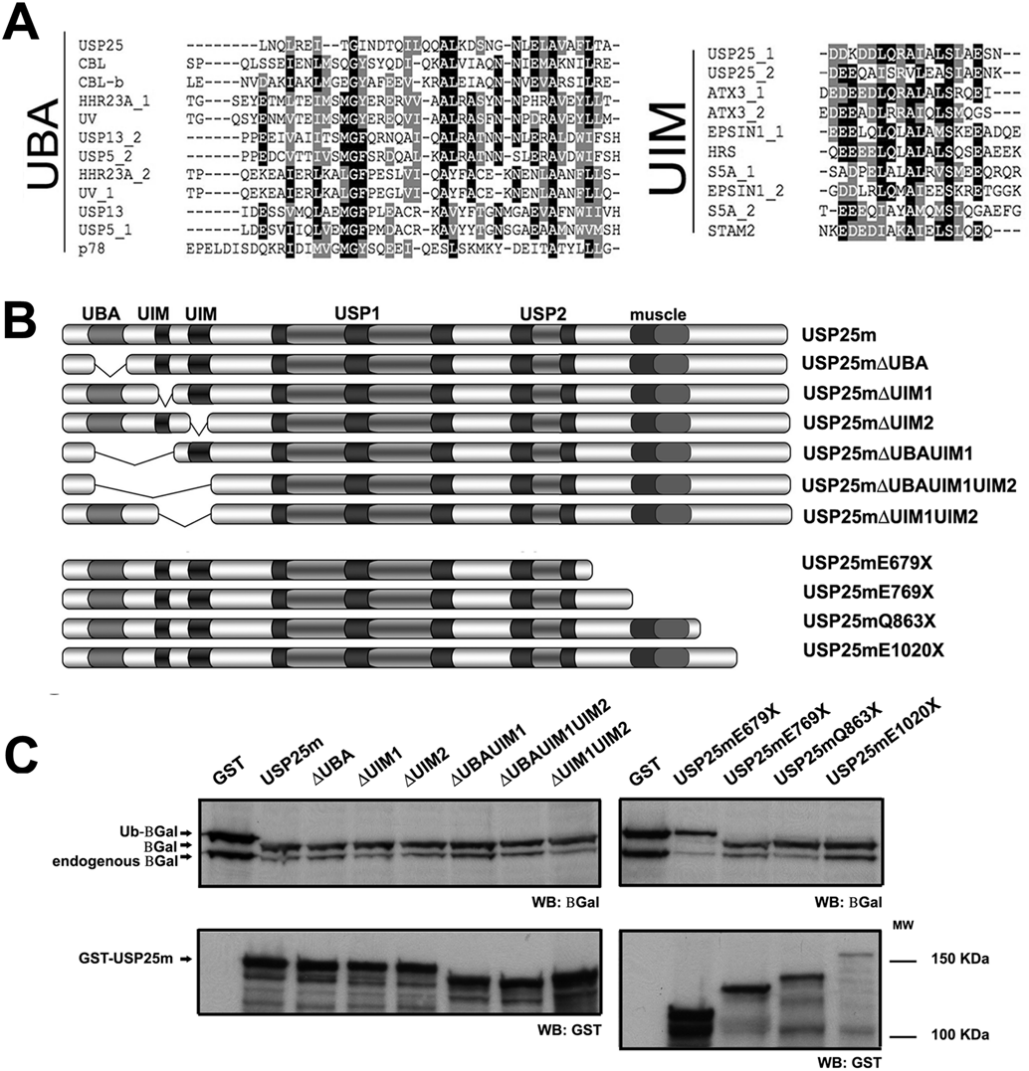
Localization of the USP25m UBDs and analysis of their contribution to the deubiquitinating activity. A. USP25m contains one UBA and two UIM (USP25_1, USP25_2) domains, as shown by alignments with other UBAs or UIMs. B. Schematic representation of the USP25m C-terminal and UBD deletion mutants: ΔUBA (Δ19-58 aa, inclusive), ΔUIM1 (Δ96-115 aa, inclusive), ΔUIM2 (Δ121-141 aa, inclusive), ΔUBA-UIM1 (Δ19-115 aa, inclusive), ΔUBA-UIM1-UIM2 (Δ19-141 aa, inclusive), ΔUIM1-UIM2 (Δ96-141 aa, inclusive). The constructs bearing serial deletions of USP25m at the C-terminus are also shown (E679X, E769X, Q863X, E1020X). C. Deubiquitinating activity assays indicated that UBDs were not required to cleave off ubiquitin (left upper panel). The mutant USP25mE679X was unable to hydrolyze Ub from the Ub-βgal substrate, indicating that the region between the amino acids 679 and 769 was required for enzymatic activity (right upper panel). The empty GST vector and the full length USP25m were respectively used as negative and positive controls. The expression level of each USP25m mutant was comparable (lower panels).

*In silico* homology searches across several motif databases revealed three Ubiquitin Binding Domains at the N-terminus of USP25m, one UBA and two UIM signatures (Figure 2A). These domains are known to interact with ubiquitinated proteins, although they seem not to be required for catalytic activity.

To assess whether the UBA and UIM domains contribute to USP25m deubiquitinating activity, we co-expressed GST epitope-tagged deletion mutants of USP25m, which lacked one or several of the UBDs (Figure 2B), with the recombinant substrate Ub-β-Gal in *E. coli*. Under these conditions, the deubiquitinating activity-assay clearly showed that deletion of UIM1, UIM2 and UBA domains, alone or in combination, did not abolish neither diminish the USP25m DUB-activity compared to the wild type enzyme (Figure 2C, left panel).

USP enzymes are usually proteins of high molecular weight, which stretch at the N- and/or the C-terminus of the USP catalytic domains. These extensions have been proposed to be involved in substrate recognition, regulation of the catalytic activity or subcellular localization. USP25 stretches more than 450 amino acids at the C-terminus, including the muscle-specific peptide (introduced by alternative spliced exons 19a and 19b, see Figure S1). We had previously shown that this tissue-specific peptide (70 amino acids) was required for recognition and rescue from proteasome degradation of sarcomeric substrates [18], but except for this experimental evidence, the function of this long C-terminus remained unassigned. We decided to perform serial deletions by introducing STOP codons by site-directed mutagenesis at positions E679X, E769X, Q863X and E1020X. As *in silico* searches did not find any functional motif or obvious homology in this region, the positions for the STOP codons were chosen by avoiding to impair secondary structures such as alpha helices or coiled-coils (Figure 2B).

In contrast with the results obtained with the UBD mutants, the analysis of the serial truncated proteins at the C-terminus of the USP25m protein clearly showed that mutant E679X was incapable of cleaving off the ubiquitin moiety of the Ub-β-gal protein, whereas mutants E769X, Q863X and E1020X still retained the enzymatic activity (Fig. 2C right panel). Thus, even though the catalytic USP domains relevant for DUBs were present in E679X (Figure S1), the deletion of 90 amino acids between E679 and E769 completely abrogated the deubiquitinating activity of USP25. It is worth noting that *in silico* predictions showed a long coiled-coil domain in this region.

As UBDs have also been involved in shifts in subcellular localization, we asssessed whether the wild-type USP25m and UBD-deleted constructs, either in their catalytically active or inactive forms, showed different localizations. No change in the distribution pattern was observed in any condition, indicating that the UBA and UIM domains were not required for targeting USP25 to its localization (Figure S2). We also monitored Ub distribution on the same cells and ruled out a possible effect on the accumulation of ubiquitinated proteins (Figure S2), as described for other USPs [21]. Nor did the USP25 C-terminal truncated mutants show any shift in their subcellular localization, as they all remained cytosolic in transient transfections on cultured cells (data not shown).

### USP25m forms complexes by dimerization/oligomerization

**Figure 3:**
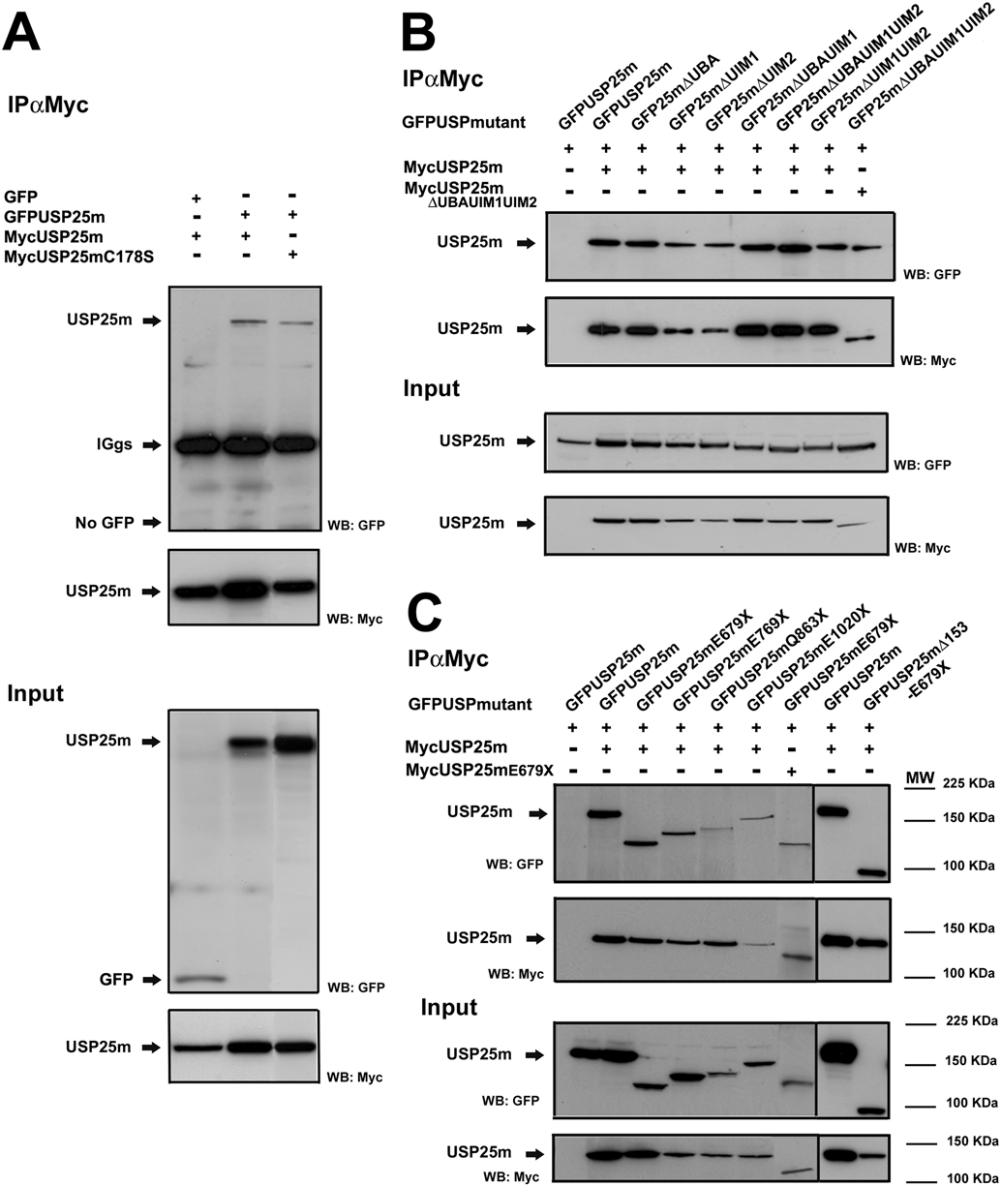
Dimerization/oligomerization of USP25m. A. Coimmunoprecipitation assays after co-expressing two differently tagged forms (cMyc- or GFP-) of either the wild-type USP25m or the C178S mutant, showed that USP25 dimerized *in vivo* (upper panel). The catalytic Cys was not required for interaction. The empty GFP vector was used as a negative control. B. The same co-immunoprecipitation experiment co-expressing the cMyc-USP25m with each of the UBD deletion mutants fused to GFP showed that none of the UBDs was critical for dimerization or formation of the complex. Last lanes of the panels correspond to the co-immunoprecitation of the two mutants bearing the deletion of the 3 UBD domains (Δ19-141, inclusive). Single transfection with the GFP-USP25m construct was used as a negative control. C. The same assays using the constructs with serial deletions of the C-terminal region of the enzyme showed that the C-terminus was not required for dimerization of USP25m. The last lane of the panels at the left corresponds to the cotransfection with two differently tagged E679X mutants. Single transfection with the GFP-USP25m construct was used as a negative control (first lane). The separated panels at the right correspond to the co-immunoprecipitation of the double mutant USP25m bearing the deletion of the first 153 amino acids and truncated at residue 679 (USP25mΔ153-E679X) with the wild-type USP25m, and their positive control.

The dynamic nature of the Ub-pathway requires the formation of complexes in which enzymes and cofactors are transiently recruited, not only E2 and E3 ligases but also DUBs [22]–[24]. We explored whether USP25m was able to dimerize/oligomerize. To this end, we used two tags, c-Myc and GFP, fused to the wild-type USP25m protein and each of the deletion USP25m mutants, respectively. Co-immunoprecipitation assays showed the interaction between the cMyc- and GFP-tagged USP25 proteins, indicating that USP25 formed homodimeric or oligomeric complexes *in vivo* (Figure 3A). The catalytically inactive enzyme, as well as all the UBD deletion mutants, also dimerized (or oligomerized) (Figure 3A and 3B). Similar results were obtained when assaying the C-terminal mutants (Figure 3C). A double mutant USP25mΔ153-E679X (in which the first 153 amino acids have been deleted, and the protein is truncated at amino acid 679) could also oligomerize (Figure 3C). Therefore, neither the UBDs nor the C-terminus of USP25m were required for this interaction. Taken together, these results suggested that the region between amino acids 153 to 679, which contained the USP domains and was not deleted in any construct, was relevant for dimerization/oligomerization.

Native gel electrophoresis followed by western blot immunodetection confirmed that USP25 was included in high molecular weight complexes (>250 kDa, data not shown). As non-denaturing conditions were used to detect protein complexes, the dimerization (oligomerization) of USP25 could either be direct or require some other substrate/partners.

### USP25m was ubiquitinated and autodeubiquitinated, and the target residue for ubiquitination *in vivo* is K99

**Figure 4:**
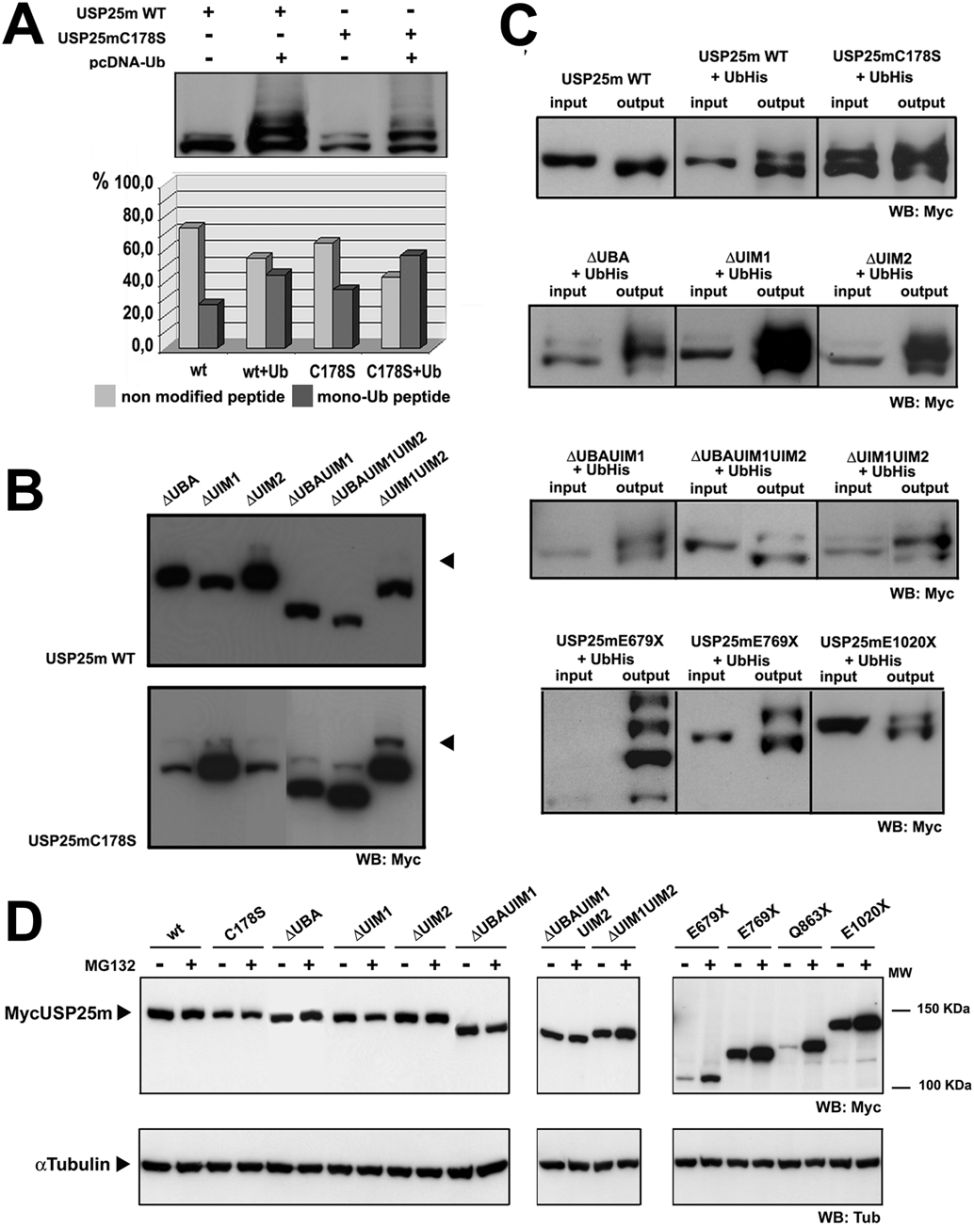
USP25m is ubiquitinated and autodeubiquitinated. A. Immunodetection of cell lysates expressing Myc-tagged USP25m showed one additional high molecular-weight band. This band was stronger when co-expressing His(6x)-Ub, suggesting that it corresponded to mono-ubiquitinated USP25m. The high molecular weight bands were stronger when co-expressing His(6x)-Ub and the catalytically inactive mutant USP25mC178S. The lower histogram shows the percentage of non-modified versus mono-Ub-conjugated USP25m. B. The same experiment was performed co-expressing His(6x)-Ub with all the UBD USP25m deletion mutants, in combination or not with the C178S mutation. Again, the ubiquitinated band was much visible in the C178S version of the mutants. C. Ni^2+^ pull-down assays to purify His(6x)Ub-conjugated proteins confirmed that USP25m was ubiquitinated. All the mutant constructs were tested, confirming that monoubiquitination (and multi- or poly-ubiquitination) did not depend on UBDs, neither on the presence of the C-terminus. The ratio output/input is 4. (Output samples were eluted at pH 4.5, which could account for the slight variation in the apparent protein molecular weight compared to inputs). D. Protein stability of the USP25m full-length and mutant constructs. Cells were grown in standard conditions (−), or treated with MG132 (+). Immunodetection of α-tubulin was used as a loading control.

Many E3 ligases and some DUBs undergo post-translational modifications, such as ubiquitination or sumoylation, which modulate the recognition of their substrates [25]. Of particular interest was to determine whether USP25m was modified by ubiquitin, given the deubiquitinating activity of the enzyme and the fact that both, mono- and poly-ubiquitination have been widely reported to regulate enzyme function. We investigated the USP25m ubiquitination status in HEK293T cells transiently co-transfected with His(6x)-Ubiquitin and Myc-tagged USP25m constructs. Immunodetection of USP25m showed an additional higher molecular weight-band (Figure 4A), around 25% of the total USP25m. This band was much weaker in lysates of cells that did not over-express the Ub construct, indicating that only a fraction of USP25m was ubiquitinated *in vivo* under our experimental conditions. Unexpectedly, the expression of the USP25m catalytically inactive form produced a much stronger high molecular-weight band (Figure 4A, lanes 3–4), which amounted to 60% of total USP25m when co-transfected with the Ub construct (Figure 4A, histogram). The fact that the proportion of modified enzyme was increased in the catalytically inactive C178S mutant, strongly indicated that the wild-type enzyme is able to autodeubiquitinate. Taken together, these results indicate that USP25m is mono-ubiquitinated *in vivo*, and that the enzyme may revert this modification by autodeubiquitination.

To examine the possible involvement of UIMs in USP25m ubiquitination [26], [27], we assayed the ubiquination state of the UBD deletion constructs. The deletion of any UIM and UBA domains, or their combination, prevented, at least partially, USP25m mono-ubiquitination (Figure 4B, upper panel). The mono-ubiquitinated bands were more apparent if the catalytically inactive forms of the deletion constructs were used (Figure 4B, lower panel). In all deletions and constructs the proportion of modified protein was clearly lower than that of the full-length USP25m.

To further study USP25m ubiquitination, we performed a Ni^2+^ pull-down assay in cells co-expressing the different USP25 mutants together with His-tagged Ubiquitin. We recovered ubiquitinated USP25m proteins in all UBD deletion mutants (Figure 4C). We also tested the C-terminus deleted USP25m forms; all of them showed intense high molecular-weight bands indicative of mono- and possible multi- or poly-ubiquitination. Noticeably, when testing the mutant that lacked the coiled coil region (E679X), most of the protein was Ub-modified. This result supported the proposed autodeubiquitination activity, as this mutant was catalytically inactive. To discern whether this multiple band pattern was caused by poly-ubiquitination to tag the deleted USP25m proteins to proteasome degradation, we performed an assay of protein stability with the proteasome inhibitor MG132 (Figure 4D). USP25m full-length as well as the UBD deletion mutants were stable at 16 hours treatment, supporting mono- and multi-Ub modification. In contrast, the protein levels of the C-terminal deletion mutants were clearly increased when the proteasome was inhibited, indicating poly-ubiquitination (Figure 4D), therefore the most C-terminal region is required for USP25 estabilization.

To identify the lysine residue involved in the mono-ubiquitination, we co-transfected cells with the mutant USP25mC178S with His(6x)Ub, enriched the lysate in USP25m forms by immunoprecipitation with an anti-cMyc antibody, and analysed the obtained bands by LC-ESI-QTOF mass spectrometry. One Ub-modified peptide appeared consistently, indicating that K99 was the most likely acceptor site (Table 1). This lysine is located at the beginning of UIM1 and most interestingly, had been previously reported to be the main acceptor for USP25 sumoylation, suggesting a dual regulatory role for this residue. Given that deletion of UIMs, although clearly diminishing USP25 ubiquitination, did not completely preclude it, other less preferential sites might become alternative acceptor sites for ubiquitination.

**Table 1:** Peptide sequences obtained by mass-spectrometry of enriched ubiquitinated USP25mC178S showed that Lys99 is the preferential site for monoubiquitination.

Peptide (+Ubiquitin modification)	Protein identified
KYVDPSR	USP25
TPTEVWR	USP25
YNDIAVTK	USP25
AIKLEYAR	USP25
YLSYGSGPK	USP25
TEIENDTR	USP25
DSRNPYDR	USP25
FLAVGVLEGK	USP25
VLEASAIAENK	USP25
TLLEQFGDR	USP25
YLFALLVGTSK	USP25
AVEILKDAFK	USP25
HQQTFLNQLR	USP25
AEEETDEEKPK	USP25
AQFLIQEEFNK	USP25
LEFPQVLYLDR	USP25
FEFNQALGRPEK	USP25
ETGITDEEQAISR	USP25
LAQEDTPPETDYR	USP25
DSNGNLELAVAFLTAK	USP25
LNEQAAELFESGEDR	USP25
ETGPQLVGIETLPPDLR	USP25
IHNKLEFPQVLYLDR	USP25
SGQEHWFTELPPVLTFELSR	USP25
LRESETSVTTAQAAGDPEYLEQPSR	USP25
YISVGSQADTNVIDLTGDDKDDLQRA (+GlyGly)	USP25 (Ubiquitinated)
EGIPPDQQR	Ubiquitin
ESTIHLVLR	Ubiquitin
IQDKEGIPPDQQR	Ubiquitin
TITLEVEPSDTIENVK	Ubiquitin

Taken together, our results strongly suggest that: i) USP25m was ubiquitinated and underwent autodeubiquitination, ii) UIM1, UIM2 and UBA domains promoted, but were not strictly required for monoubiquitination, iii) the C-terminal region is relevant for the protein stability and, when deleted, USP25m is polyubiquitinated and targeted for proteasome degradation, and iv) the preferential target lysine for ubiquitination is K99.

Given that USP25 was also reported to be a target for SUMO [19], we assayed other potential USP25 post-translational modifications. The *in vitro* assay showed that indeed SUMO-1 and SUMO-2 were conjugated to USP25m. In addition, our results in cultured cells revealed that USP25m was phosphorylated (in Tyr and Ser/Thr residues) and acetylated, and that these modifications were independent of USP25m catalytic activity, as the wild-type protein and the inactive mutant were similarly modified (Figure S3). Further work is needed to assess whether these modifications modify the catalytic activity of USP25.

### UBDs modulate USP25m substrate recognition

Although the targets of most DUBs are unkown, USP25 is a DUB that specifically recognizes and binds its substrates in physiological conditions. We previously reported that the muscle-specific isoform USP25 interacted with MyBPC1, and that the DUB activity of USP25m rescued this substrate from proteasome degradation. This recognition was highly specific and depended on the peptide encoded by the muscle-specific exons 19a and 19b, as the ubiquitous USP25 isoform was unable to rescue this substrate [18].

**Figure 5:**
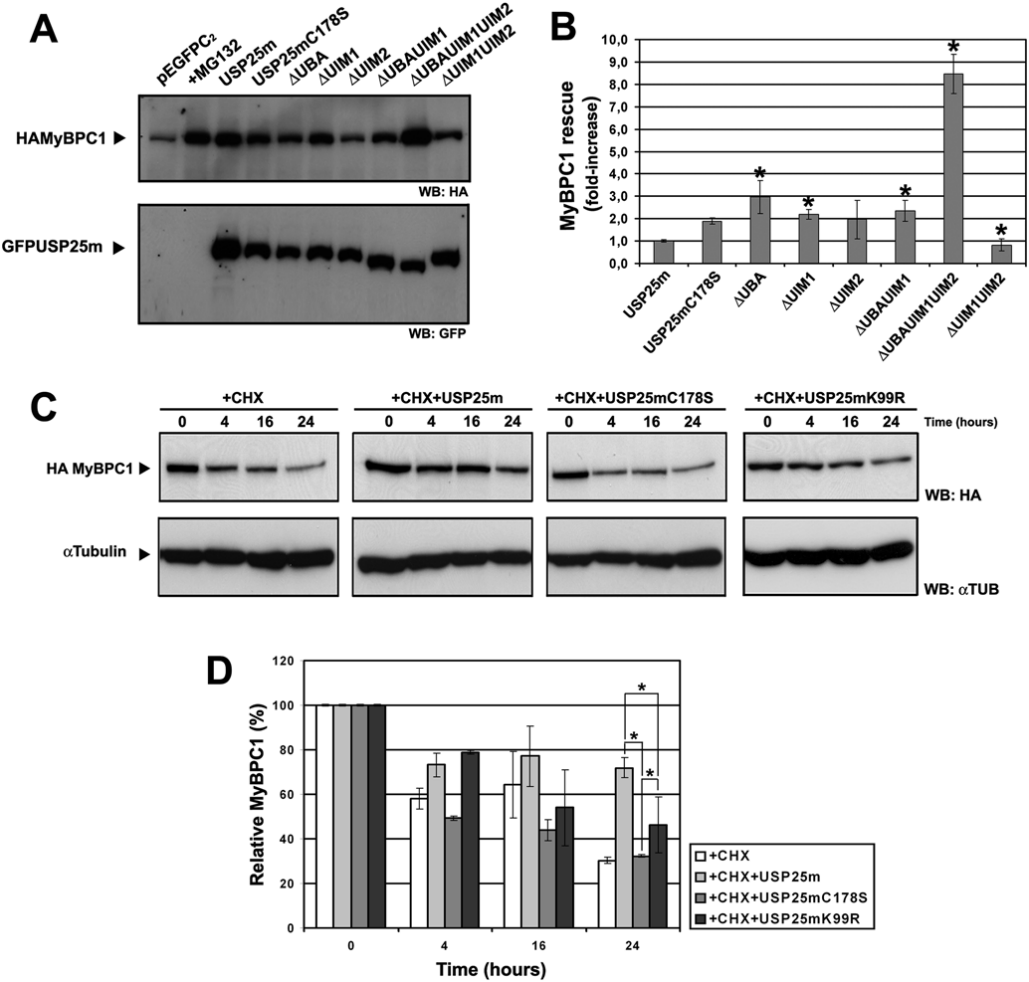
UBDs modulate substrate recognition by USP25m and K99 is the key regulatory residue. A. MyBPC1 is differentially rescued from proteasome degradation depending on the presence of the distinct UBDs. Transfection of MyBPC1 with the empty GFP vector was used as the negative control, and addition of MG132 was used as a positive control. B. Relative quantification of the MyBPC1 rescue by different USP25m mutants. α-tubulin was used for normalization of protein concentration (data not shown) and USP25m expression levels were used to normalize for transfection efficiency. The rescue achieved by the wild-type USP25m was considered as the reference (value of one). At least three different replicates were used for quantification. Asterisks indicate statistical significance (p<0.05, Mann-Whitney test). C. The catalytically inactive C178S and the K99R mutants behaved similarly and are unable to rescue MyBPC1 from proteasome degradation in a time-course experiment when new protein synthesis is inhibited. The rescue achieved by expression of the wild-type USP25m was used as a control. D. The MyBPC1 levels (normalized by α-tubulin expression) were quantified and expressed relatively to those observed at time 0 h (30 h post-transfection, before cycloheximide treatment), which were considered 100%. The values corresponded to a minimum of three different replicates in several independent experiments. Asterisks indicate statistical significance (p<0.05, Mann-Whitney test). CHX- cicloheximide.

Given the reported relevance of UBDs in the regulation of protein folding and modular domain interactions, we were prompted to test the effect of the absence of UBA and/or UIM domains of USP25m in the rescue of MyBPC1 from proteasome degradation. As a positive control, the expression of the wild-type USP25m rescued MyBPC1 to the levels attained with the MG132 proteasome inhibitor (Figure 5A). Interestingly, all the UBD mutants recognized and rescued MyBPC1 from proteasome degradation, although with varying efficiency (compare lane 1 with lanes 5 to 10 in Figure 5A). The activity of the mutants in rescuing MyBPC1 was compared, considering the rescue by the wild-type USP25m as the reference (Figure 5B). Single deletion of the UIM2 did not significantly affect the recognition and rescue of MyBPC1, whereas the deletion of UIM1 (2-fold) or the UBA domains (3-fold) considerably increased the levels of MyBPC1. The double deletion of the UIM1UIM2 decreased the rescue of the substrate. Interestingly, the deletion of the three UBD domains, increased the rescue of MyBPC1 up to 8-fold, indicating that UBDs are not strictly required for MyBPC1 recognition and rescue, but rather they are involved in the enzyme catalytic regulation and/or access to the substrate, probably as a response to cellular requirements. Of note, this deletion not only included the UBDs, but also the SIM domain (SUMO-interacting motif) and the preferential ubiquitin/SUMO target, the residue K99.

### Mutation of K99 inhibits USP25m activity

As aforementioned, previous reports showed that sumoylation of USP25 occurred at K99, and this modification inhibited USP25m deubiquitinating activity on tetraubiquitin chains [19]. We have also showed in this work that this residue was also the preferential target for ubiquitination. Given that the two modifications are mutually exclusive, we hypothesized that ubiquitination in K99 would cause the opposite effect, activating USP25. Mutation of K99 to arginine would eliminate the preferential sites for both, sumoylation and ubiquitination, and we could then explore the effect of this mutation in USP25m activity directly on MyBPC1, a physiological substrate. As controls, we used both the wild-type enzyme and the catalytically inactive mutant C178S.

As expected, the USP25mC178S was not able to rescue MyBPC1 in a time-course experiment, whereas the expression of the wild-type USP25m raised the half-life of MyBPC1, as its levels were steadily maintained through time when protein synthesis was inhibited (Figure 5C and [18]). Remarkably, USP25mK99R was not able to rescue MyBCP1 from proteasome degradation, as the MyBPC1 levels steadily declined. After 16 h treatment with cicloheximide, the MyBPC1 levels were already decreased to 50%, and after 24 h, the levels of MyBPC1 were much lower than those obtained by the wild-type enzyme, although higher than those obtained by the catalytically inactive mutant (statistical significance p<0.05, Mann-Whitney test) (Figure 5D). If the K99 mutation merely prevented sumoylation, and sumoylation inhibited USP25, then we should have expected an increase of DUB activity for the USP25mK99R mutant. However, the fact that the K99R mutant was less effective in rescuing its substrate indicated that the alternative modification of this lysine, namely ubiquitination, resulted in USP25m activation.

## Discussion

As DUBs are the least known members of the UPS, we studied the physiological function of USP25 by domain dissection. We particularly focussed in the three predicted UBDs, as these motifs are usually clustered in the same protein and confer subtle differences in the interaction with ubiquitinated substrates. By generating serial and combinatorial deletions, we assessed USP25 protease activity on a recombinant substrate, and showed that all UBD deletion mutants were catalytically active. We concluded that these domains were not strictly required for ubiquitin recognition or the deubiquitinating activity.

Increasing evidence support that ubiquitin-pathway enzymes (E2–E3 ligases, and more recently, DUBs) form cooperative complexes [22]–[24]. Our results indicate that USP25 was able to dimerize/oligomerize. Although most cysteine proteases have not been reported to require oligomerization for catalysis, crystallographic data showed homodimerization for another USP, USP8 [28], providing further grounds for USP25 dimerization. This interaction could occur before or upon substrate binding, and thus provide a means of regulation. In this context, a plausible explanation for the formation of dimers would be USP25 intermolecular autodeubiquitination (see the model below). In addition, dimers/oligomers could facilitate the progressive deubiquitination of a multi- or poly-ubiquitinated substrate, or alternatively, alter the interfaces displayed for substrate recognition.

One of the reported functions of UBA and UIM sequences is the promotion of ubiquitination of the protein in which they are embedded, thus facilitating autoregulation [29]. In the ubiquitin pathway enzymes, feedback self-regulation loops become more complex, as E3 ligases promote their autoubiquitination and DUBs, their autodeubiquination, under certain physiological stimuli [30], [31]. Evidence for mono- or multi- ubiquitination of USP25m was gathered as a faint high molecular weight band (around 8 kDa larger than that of USP25m) after co-expression of USP25m and ubiquitin. Notably, this band was significantly enriched in lysates of the catalytically inactive USP25mC178S, further suggesting both, that it corresponded to monoubiquitinated forms, and that USP25 catalyzed its own deubiquitination. Mass spectrometry of enriched USP25mC178S samples indicated that USP25 was conjugated to ubiquitin, and that K99 (located in UIM1) was the main target residue. Deletion of the UBDs reduced considerably, but did not abrogate ubiquitination of USP25m, as in all cases ubiquitinated forms were recovered. Thus, the USP25 UBDs, in particular UIM1, enhanced the ubiquitination state of the protein by either providing the preferred lysine residue, directly recruiting E2 or E3 ligases, or both. In the deletion mutants, ubiquitination might take place in alternative lysines with less efficiency. In fact, the use of preferential and alternative lysine residues for mono-ubiquitin conjugation had been previously reported [32].

Concerning the ubiquitination state and fate of the wild-type protein and the UBD mutants, we surmised that it corresponded mainly to mono- and multi- ubiquitinated forms, not related to protein degradation, as they were stable through time under our conditions. In contrast, the modification of the C-terminus mutants was compatible with polyubiquitination, as their protein levels were increased when the proteasome was inhibited, pointing to the relevance of the last 106 amino acids in USP25m stability. Polyubiquitination did not appear to be related to the catalytic activity of USP25m as: i) truncated mutants E1020X, Q863X and E769X were enzymatically active but degraded by the proteasome, and ii) of the two catalytically inactive E679X and C178S, the former was polyubiquitinated and degraded, whereas the latter was monoubiquitinated and this modification was not related to degradation. Therefore, autodeubiquitination does not seem to be required for USP25m stability.

Finally, we assessed the contribution of the UBD deletion mutants to the recognition of the USP25m specific substrate MyBPC1, considering that the requirements for the interaction with a specific physiological substrate might be different from those of a synthetic polyubiquitin substrate. None of the UBDs was critical for enzyme-substrate interaction, as all the mutants rescued the substrate from proteasome degradation. However, the effects were distinct depending on the domains deleted or preserved. The analysis of the contribution of the single and combined domains suggest that the UBA domain negatively modulated the USP25 function mainly by interaction with the UIM1 domain. The effect of the two UIM domains on the substrate rescue appeared to fit an additive/synergical mode of action. Deletion of the three UBDs would effectively remove all these regulatory domains, including those involved in SUMO modification and the target K99. Given that the overexpression of this UBD-deleted USP25 construct caused increased rescue of MyBPC1, we interpreted that the lack of these regulatory domains allowed USP25m free (non-regulated) access to its substrate. UBDs then would mostly contribute to the enzyme regulation in response to cellular requirements rather than to strict substrate recognition.

### Model for USP25m regulation: the dual role of K99

**Figure 6:**
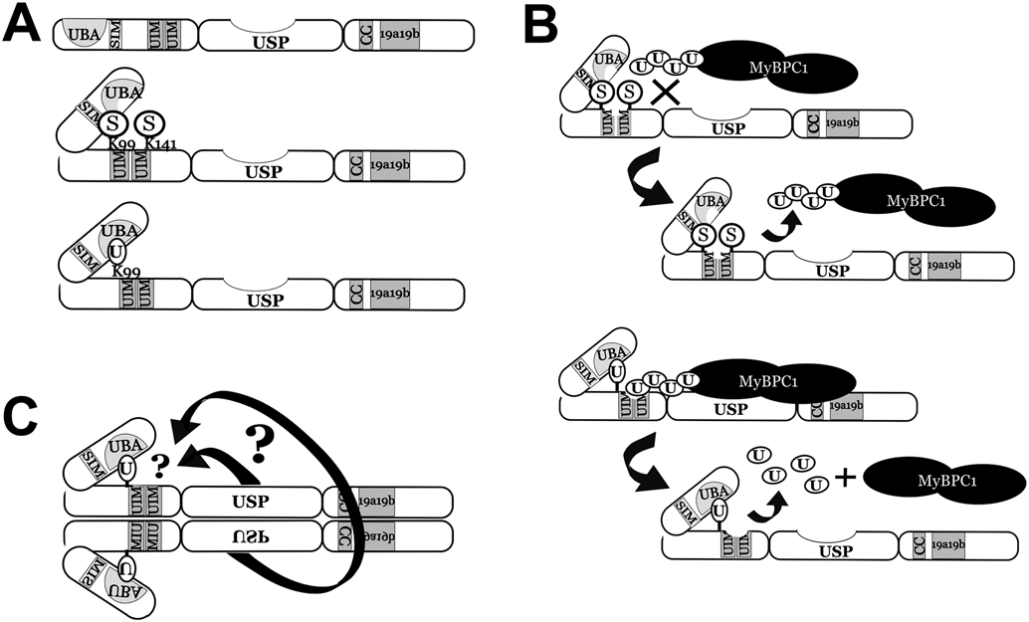
Model of the USP25m activity regulation upon specific substrates. A. Post-translational modifications of USP25m concerning monoubiquitination in Lys99 (K99) and sumoylation in K99 and K141. U: Ubiquitin; S: SUMO; UBA: Ubiquitin associated domain; SIM: SUMO interacting motif; UIM: Ubiquitin interacting motif; USP: Ubiquitin specific protease catalytic domains; CC: coiled coil domain; 19a19b: peptide encoded by the muscle-specific exons. B. Model on the regulation of USP25m activity through alternative and mutually exclusive conjugation of SUMO (inhibiting) and ubiquitin (activating) on the same lysine residue (K99) (see discussion). C. The dimerization of USP25m might be relevant to the regulation of the enzymatic activity as autodeubiquitination could occur either intra- or inter-molecular in the dimer.

Ubiquitin and SUMO pathways may engage in cross-talk, determining opposite fates or functions of a particular substrate, and even compete for the same residues [33]. This seems to be the case for USP25 regulation. Our results together with those of other authors [19] support a combined model for the regulation of USP25 enzymatic activity and substrate recognition based on the dual role of K99 as a target for both SUMO and ubiquitin. In response to cellular requirements, USP25 would undergo several modifications, in particular, monoubiquitination at K99 or sumoylation at the same residue (Figure 6A). SUMO conjugation at K99 (and also at the secondary site K141) depends on the interaction with the proximal SIM domain, and results in inhibition of the USP25 protease activity on polyubiquitinated chains *in vitro* [19], [20]. Therefore, in physiological conditions sumoylation would impair the rescue of substrates from proteasome degradation by USP25. On the contrary, ubiquitination of K99 would result in enzyme activation by either preventing sumoylation or by allowing new interactions. The modulation of the active enzyme would depend then on the interplay between the UBA and the ubiquitinated K99 in UIM1, either intra-o or inter-molecularly (Figure 6B, for the sake of simplicity the model only shows intramolecular recognition).

Further regulation of the enzyme activity would rely on autodeubiquitination (either intra- or inter- molecularly in a dimer/complex), which would make this lysine residue available for alternative modifications, thus allowing the shift between the enzymatic activity states (Figure 6C).

According to this model, regulation and integration of cell signals would be exerted through the N-terminus of USP25, where the SIM, UBDs and the preferential sites for SUMO and ubiquitin conjugation are clustered. In this context, the deletion of the three UBDs would remove all the regulating domains of the enzyme and permit free access to the substrate, which would explain the higher rescue obtained for this mutant. Indeed, we have previously showed that the recognition of the specific substrate MyBPC1, was dependent not on UBDs but on the peptide encoded by exons 19a19b [18].

Deubiquitinating enzymes have to integrate cellular signals and promote dynamic interactions with their substrates, similarly to what occurs with E2–E3 ligases. Modification of a single target residue in USP25 by SUMO (inhibiting) or ubiquitin (activating), combined with the cluster of SIM and UBDs domains in the same molecule, provides new insights and open new avenues for the study of DUB regulation concerning substrate recognition and catalytic activity. To illustrate this statement, the USP25 closest homolog (sharing 52% of amino acid identities) is USP28 [34], a DUB involved in MYC stability as response to DNA damage [35]. USP28 displays UBA and UIM1 domains in the same location as USP25, including a conserved lysine 96 that may play a similar role in the USP28 regulation as K99 in USP25. Further work will show whether this enzyme similarity extends from structural domains to regulatory mechanisms.

## Materials and Methods

### In silico identification of USP25 structural domains

The USP25m protein sequence was analyzed using the InterPro (http://www.ebi.ac.uk/InterProScan/) and Pfam (http://www.sanger.ac.uk/ Software/Pfam/search.shtml) databases to search for functional domains. Both tools retrieved a UBA domain and two UIM domains at the N-terminus of USP25m.

### Constructs for expression of serial and combinatorial deletions as well as generation of USP25m point mutants

Mutants USP25mC178S and USP25mK99R were generated by site directed mutagenesis to serine using the QuickChange Site-Directed Mutagenesis Kit (Stratagene). Expression constructs with the full-length USP25m cloned in pGEX-4-T1 (GE Healthcare), pcDNA3 (Invitrogen) and pEGFP-C2 (Clontech), were used to generate by PCR the UBD deletion mutants of USP25m (ΔUBA, ΔUIM1, ΔUIM2, ΔUBAUIM1, ΔUBAUIM1UIM2 and ΔUIM1UIM2). The Accuprime *Taq*DNA Polymerase High Fidelity (Invitrogen) was used to avoid possible mutations. Serial deletions of the C-terminal USP25 region were generated by introducing a STOP codon by site-directed mutagenesis in positions E679X, E769X, Q863X and E1020X, and cloned into pGEX-4-T1 (GE Healthcare), pcDNA3 (Invitrogen) and pEGFP-C2 (Clontech). Integrity of the clones was verified by sequencing.

### Ubiquitin-specific protease activity assay

The ubiquitin-specific protease activity of USP25m and of all the mutant constructs was analyzed as described elsewhere [17]. Briefly, the corresponding cDNAs cloned in-frame in pGEX-4-T1 Amp^R^ downstream the glutathione-S-transferase (GST) gene, and the plasmid pACY184 Cm^r^ expressing Ub-Met-β-gal (a kind gift from Dr. M. Hoschtrasser) were co-transformed in *E. coli* XL1blue. Clones resistant to both Amp and Cm were grown and induced for 3 hours with isopropyl-β-thiogalactopyranoside (final concentration 1 mM). Total protein extracts were analyzed by western blot using anti-β-galactosidase mouse monoclonal antibody (dilution 1∶1000, Sigma-Aldrich) and anti-GST monoclonal (dilution 1∶1000, Santa Cruz Biotechnology).

### Co-immunoprecipitation assays

HEK293T cells were seeded on 100 mm tissue culture dishes (2×10^5^ cells/dish). After 16 h, cells were transiently co-transfected with cMyc-USP25m and GFP-USP25m, either full-length or the deletion mutants at the N- and C- terminus, using Lipofectamine 2000 (Invitrogen). Cells were collected 42 h postransfection, resuspended in lysis buffer (0.5% Nonidet P-40, 50 mM TrisHCl pH 7.5, 1 mM EDTA, 150 mM NaCl and protease inhibitor cocktail (Roche) and lysed by sonication. Protein extracts were recovered after removal of cellular debris by centrifugation, incubated at 4°C with 2 µg of anti-cMyc mAb (Santa Cruz Biotechnology) during 4 hours with end-over-end mixing. The protein-antibody complexes were removed with 1 hour incubation at 4°C with protein G-Sepharose beads (Amersham GE-Healthcare). After washing, bound proteins were eluted from the beads by boiling 5 min with protein loading buffer, loaded onto 8% SDS-PAGE gels and analysed by Western Bloting using anti-GFP pAb (1∶1000, Santa Cruz Biotechnology), anti-cMyc mAb (1∶1000, Santa Cruz Biotechnology).

### *In vivo* assessment of ubiquitin conjugation

Human embryonic kidney (HEK)293T cells were plated (2×10^6^) in 10 cm Petri dishes. After 12 hours, they were co-transfected with 6 µg of a construct expressing His(6x)-Ub (kindly provided by Dr. M. Rodriguez) and 6 µg of pcDNA-Myc-USP25m, or pcDNA-Myc-USP25mC178S, either in their full length version or with the UBD-deletion mutants (ΔUBA, ΔUIM1, ΔUIM2, ΔUBAUIM1, ΔUBAUIM1UIM2 and ΔUIM1UIM2), or the C- terminal (679X, 769X and 1020X) deletion mutants, using Lipofectamine 2000 (Invitrogen). Forty-eight hours post-transfection, cells were washed with PBS and resuspended in 1.4 ml of denaturing lysis buffer pH 8.0 (50 mM sodium-phosphate buffer pH 8.0, 8 M urea, 300 mM NaCl, 0.5% Triton X-100, with 10 mM iodoacetamide and 10 mM NEM, freshly added) and stored at −80°C. Cleared cell lysates were loaded onto 8% SDS-PAGE gels and analyzed by Western blotting with anti-cMyc monoclonal antibody (1∶1000, Santa Cruz Biotechnology).

For the Ni^2+^ pull-down assay, cell lysates obtained as described above, were incubated with 80 µl of His-Select Nickel Affinity Gel (Sigma-Aldrich) during 3 h at room temperature. After 3 washes with the following buffer at pH 6.3 (50 mM sodium-phosphate buffer pH 6.0, 8 M urea, 300 mM NaCl), samples were eluted by boiling 5 minutes in 100 µl of protein-loading buffer (60 mM TrisHCl pH 6.8, 10% glycerol, 2% SDS, 0.1% bromophenol blue and 10% β-mercaptoethanol) and loaded onto 8% SDS-PAGE gels. After blotting, the proteins were detected by Western as stated above.

For further assessment of ubiquitination, cell lysates were incubated at 4°C with 2 µg of anti-cMyc mAb (Santa Cruz Biotechnology) during 4 hours with end-over-end mixing. The protein-antibody complexes were removed by one hour incubation at 4°C with protein G-Sepharose beads (Amersham GE-Healthcare). After thorough washing, bound proteins were eluted from the beads by boiling 5 min with protein loading buffer and loaded onto 8% SDS-PAGE gels. Bands were excised after Coomassie-Blue R250 staining and trypsinized. Tryptic peptides were analyzed in MALDI-TOF/TOF (4700 Proteomics Analyzer, Applied Biosystems) and/or in LC-ESI-QTOF (Q-TOF Global, Micromass-Waters) mass spectrometers and submitted using a MASCOT database search engine against non-redundant NCBi or SwissProt databases.

### MyBPC1 rescue assays and protein stability of USP25m constructs

HEK293T cells were seeded on 24-well plates (2×10^5^ cells/well). After 12 hours, cells were transiently co-transfected with constructs expressing HA-MyBPC1 and GFP-USP25m (full-length, or the corresponding deletion mutants), using Lipofectamine 2000 (Invitrogen). When stated, the proteasome inhibitor MG132 (10 µM, Sigma) was added to the medium during the last 16 hours of culture and collected 48 hours postransfection. Inhibition of new protein synthesis was achieved by adding cycloheximide (CHX, 150 µmol/ml, Sigma) to the medium 30 h postransfection and cells were collected immediately or after 4, 16 or 24 hour treatment. Cells were washed with PBS and recovered with 250 µl of protein loading buffer. Samples were loaded onto 8% SDS-PAGE gels and analyzed by western blotting using anti-HA monoclonal antibody (1∶1000, Santa Cruz Biotechnology) and anti-GFP polyclonal antibody (1∶1000, Santa Cruz Biotechnology) to assess the expression levels of MyBPC1 and USP25m, respectively. Films were scanned and quantified using QuantityOne software (Bio-Rad).

## Supporting Information

Figure S1 In silico predictions of functional domains and secondary structure of USP25m. Localization of the predicted Ubiquitin Binding Domains (one UBA and two UIMs), the catalytic deubiquitinating domains (USP), the peptides encoded by the muscle-specific alternatively spliced exons (19^a^ and 19b), and several potential sumoylation sites and phosphorylation sites. In silico searches used the InterPro (http://www.ebi.ac.uk/InterProScan) and Pfam (http://www.sanger.ac.uk/Software/Pfam/search.shtml) databases. The red star indicates the position of the catalytic cysteine mutated on the inactive mutant. Blue arrowheads indicate the C-terminal truncation mutants. The lysines that can be conjugated to either SUMO (K99 and K141) or ubiquitin (K99) are highlighted. The SUMO Interacting Motif (SIM), which partially overlaps the first UIM is also indicated (from Meulmeester et al., 2008).
(6.61 MB TIF)

Figure S2 UBDs do not alter USP25m subcellular localization. USP25m localization was monitored by immunohistochemistry using a polyclonal antibody against USP25. Localization of full length USP25m and deletion mutants is predominantly cytosolic, with certain accumulation in the perinuclear region. Transfection of full length USP25m, or the deletion mutants, does not affect distribution of Ub, as assessed by immunodetection with an anti Ub antibody.
(2.19 MB DOC)

Figure S3 USP25 is sumoylated, phosphorylated and acetylated. A. USP25m is sumoylated. USP25m and all the UBD deletion mutants display an extra higher molecular weight band (asterisk) after in vitro sumoylation assays with SUMO-1 (middle lanes) and SUMO-2 (right lanes). In the case of USP25m lacking both UBA and UIM1, the band corresponding to SUMO-USP25m is weaker (two asterisks). Note that the absence of all three UBDs rendered similar levels of USP25m sumoylation to that of the full-length protein. B. USP25m is phosphorylated. Myc-tagged USP25m and USP25mC178S were immunoprecipitated with Myc antibodies and detected in Western blots with pan-anti-Phospho-Ser and pan-anti-phospho-Tyr. Bands appearing at the size corresponding to USP25m indicate that USP25m is phosphorylated both in serine(s) and threonine(s) (1st and 2nd panel, middle lane). This band also appears when expressing USP25mC178S, indicating that USP25m phosphorylation occurs irrespectively of its catalytic activity (1st and 2nd panel, right lane). Membranes were stripped and detected with a Myc antibody to confirm that the band corresponded to USP25m (3rd panel). Immunoprecipitation inputs were assessed with antibodies against phosphorylated AKT and Myc as phosphorylation and transfection controls respectively (4th and 5th panels). C. USP25m is acetylated. Myc-tagged USP25m and USP25mC178S were immunoprecipitated with Myc antibodies and detected in Western blots with pan-anti-acetylated-Lys. Bands appearing at the USP25m size indicate it is acetylated, both WT and C178S (upper panel). The same membrane was stripped and detected with anti-Myc to confirm the identity of the bands (2nd panel). Immunoprecipitation inputs were assessed with antibodies against acetylated p53, Myc and α-Tubulin as acetylation, transfection and loading controls, respectively (3rd, 4th and 5th panels).
(1.01 MB DOC)
