# Supplementary figures and images for: The UBA-UIM Domains of the USP25 Regulate the Enzyme Ubiquitination State and Modulate Substrate Recognition
Source: PLoS One. 2009 May 15;4(5):e5571. doi: 10.1371/journal.pone.0005571 (PMC2679190; doi:10.1371/journal.pone.0005571)

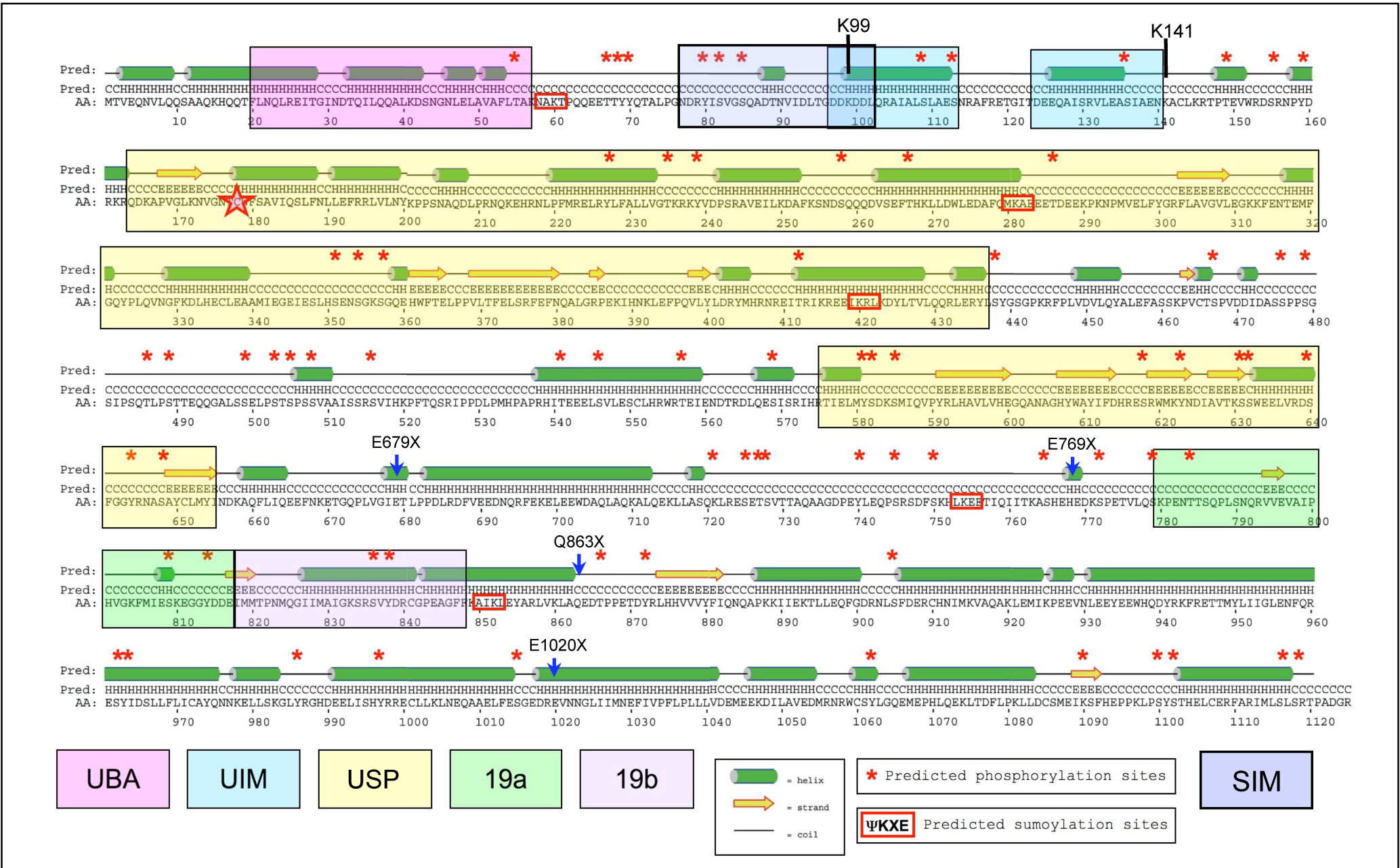

Supplement: Figure S1 — In silico predictions of functional domains and secondary structure of USP25m. Localization of the predicted Ubiquitin Binding Domains (one UBA and two UIMs), the catalytic deubiquitinating domains (USP), the peptides encoded by the muscle-specific alternatively spliced exons (19a and 19b), and several potential sumoylation sites and phosphorylation sites. In silico searches used the InterPro (http://www.ebi.ac.uk/InterProScan) and Pfam (http://www.sanger.ac.uk/Software/Pfam/search.shtml) databases. The red star indicates the position of the catalytic cysteine mutated on the inactive mutant. Blue arrowheads indicate the C-terminal truncation mutants. The lysines that can be conjugated to either SUMO (K99 and K141) or ubiquitin (K99) are highlighted. The SUMO Interacting Motif (SIM), which partially overlaps the first UIM is also indicated (from Meulmeester et al., 2008). (6.61 MB TIF) [file pone.0005571.s001.tif]
