# Supplementary material for: The UBA-UIM Domains of the USP25 Regulate the Enzyme Ubiquitination State and Modulate Substrate Recognition
Source: PLoS One. 2009 May 15;4(5):e5571. doi: 10.1371/journal.pone.0005571 (PMC2679190; doi:10.1371/journal.pone.0005571)
Supplement: Figure S2 — UBDs do not alter USP25m subcellular localization. USP25m localization was monitored by immunohistochemistry using a polyclonal antibody against USP25. Localization of full length USP25m and deletion mutants is predominantly cytosolic, with certain accumulation in the perinuclear region. Transfection of full length USP25m, or the deletion mutants, does not affect distribution of Ub, as assessed by immunodetection with an anti Ub antibody. (2.19 MB DOC) [file pone.0005571.s002.doc]

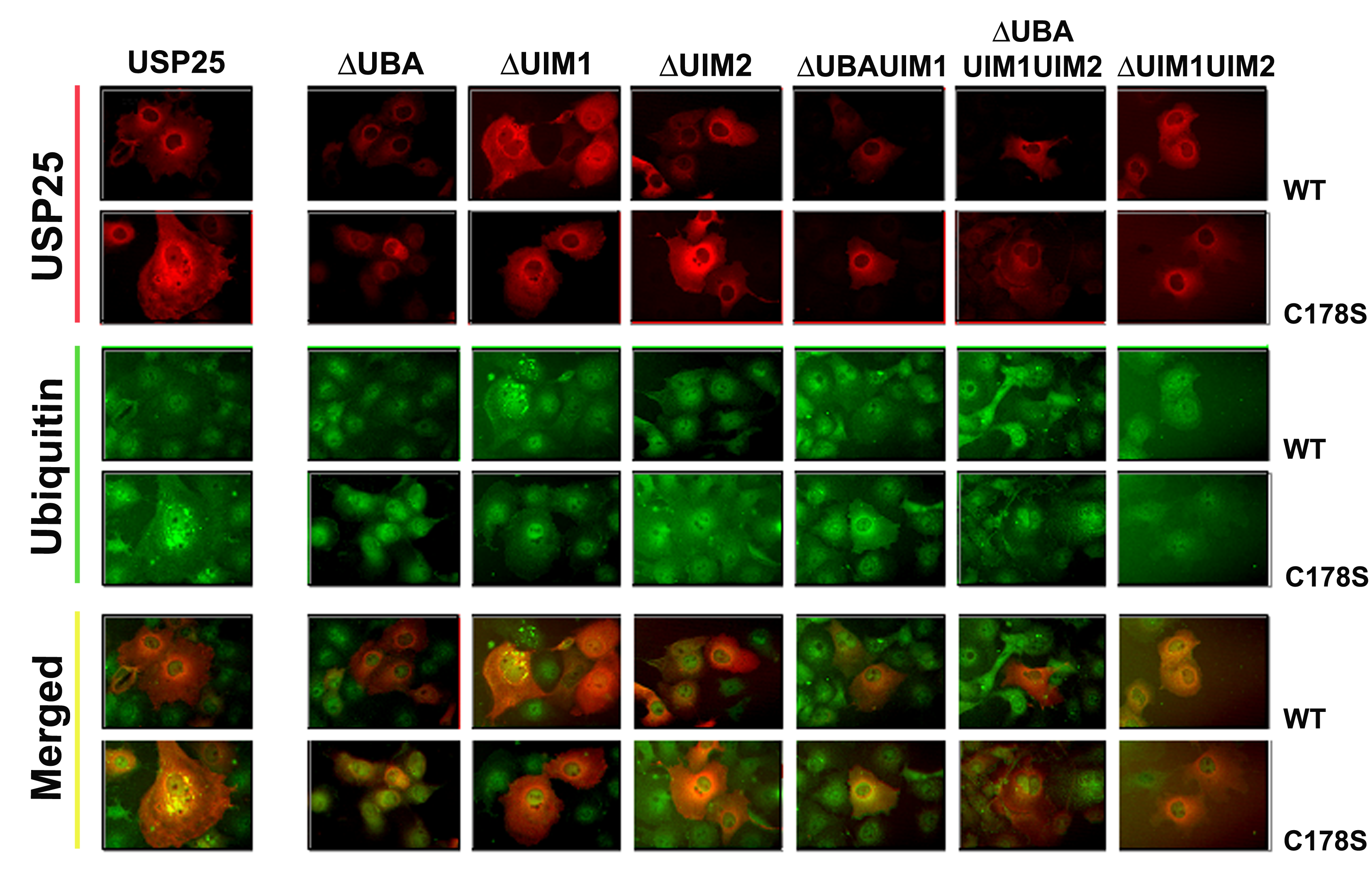


**Materials and methods**

COS-7 cells grown on glass coverslips placed in 24-well plates were transiently co-transfected with pcDNA-HA-Ubiquitin (kindly provided by Dr. Maria Masucci) and pcDNA-Myc-USP25m or pcDNA-Myc-USP25mC178S either in their full length version or in their deleted forms (ΔUBA, ΔUIM1, ΔUIM2, ΔUBAUIM1, ΔUBAUIM1UIM2 and ΔUIM1UIM2) with Lipofectamine 2000 (Invitrogen) according to the manufacturer’s instructions. When required, proteasome inhibitor MG132 (10 µM) (Biomol) was added to the medium during the last 16 hours of culture.

Forty-eight hours post transfection, cells were washed in ice-cold PBS, fixed for 20 min with freshly prepared 4% formaldehyde and blocked in 2% goat serum 1 hour at room temperature.

Samples were immunodetected with mouse monoclonal anti-Ub antibody and rabbit polyclonal anti-USP25 were diluted 1:200 in 0,05 M Tris-HCl, pH 7.4, 0.9% NaCl, 0.25% Gelatin and 0.5% Triton-X-100 overnight at 4ºC. Cells were then rinsed three times with PBS and incubated with AlexaFluor 488-conjugated anti-mouse IgG and AlexaFluor 660-conjugated anti-rabbit IgG (Molecular Probes, Invitrogen) secondary antibodies (all diluted 1:300 in the same solution than the primary antibodies) for 1 hour at room temperature. Cells were rinsed 5 times in PBS and the coverslips were mounted using Vectashield with Dapi (Vector Laboratories, Inc.). Fluorescence microscopy was performed with a Leica DMIL.
