# Supplementary material for: The UBA-UIM Domains of the USP25 Regulate the Enzyme Ubiquitination State and Modulate Substrate Recognition
Source: PLoS One. 2009 May 15;4(5):e5571. doi: 10.1371/journal.pone.0005571 (PMC2679190; doi:10.1371/journal.pone.0005571)
Supplement: Figure S3 — USP25 is sumoylated, phosphorylated and acetylated. A. USP25m is sumoylated. USP25m and all the UBD deletion mutants display an extra higher molecular weight band (asterisk) after in vitro sumoylation assays with SUMO-1 (middle lanes) and SUMO-2 (right lanes). In the case of USP25m lacking both UBA and UIM1, the band corresponding to SUMO-USP25m is weaker (two asterisks). Note that the absence of all three UBDs rendered similar levels of USP25m sumoylation to that of the full-length protein. B. USP25m is phosphorylated. Myc-tagged USP25m and USP25mC178S were immunoprecipitated with Myc antibodies and detected in Western blots with pan-anti-Phospho-Ser and pan-anti-phospho-Tyr. Bands appearing at the size corresponding to USP25m indicate that USP25m is phosphorylated both in serine(s) and threonine(s) (1st and 2nd panel, middle lane). This band also appears when expressing USP25mC178S, indicating that USP25m phosphorylation occurs irrespectively of its catalytic activity (1st and 2nd panel, right lane). Membranes were stripped and detected with a Myc antibody to confirm that the band corresponded to USP25m (3rd panel). Immunoprecipitation inputs were assessed with antibodies against phosphorylated AKT and Myc as phosphorylation and transfection controls respectively (4th and 5th panels). C. USP25m is acetylated. Myc-tagged USP25m and USP25mC178S were immunoprecipitated with Myc antibodies and detected in Western blots with pan-anti-acetylated-Lys. Bands appearing at the USP25m size indicate it is acetylated, both WT and C178S (upper panel). The same membrane was stripped and detected with anti-Myc to confirm the identity of the bands (2nd panel). Immunoprecipitation inputs were assessed with antibodies against acetylated p53, Myc and α-Tubulin as acetylation, transfection and loading controls, respectively (3rd, 4th and 5th panels). (1.01 MB DOC) [file pone.0005571.s003.doc]

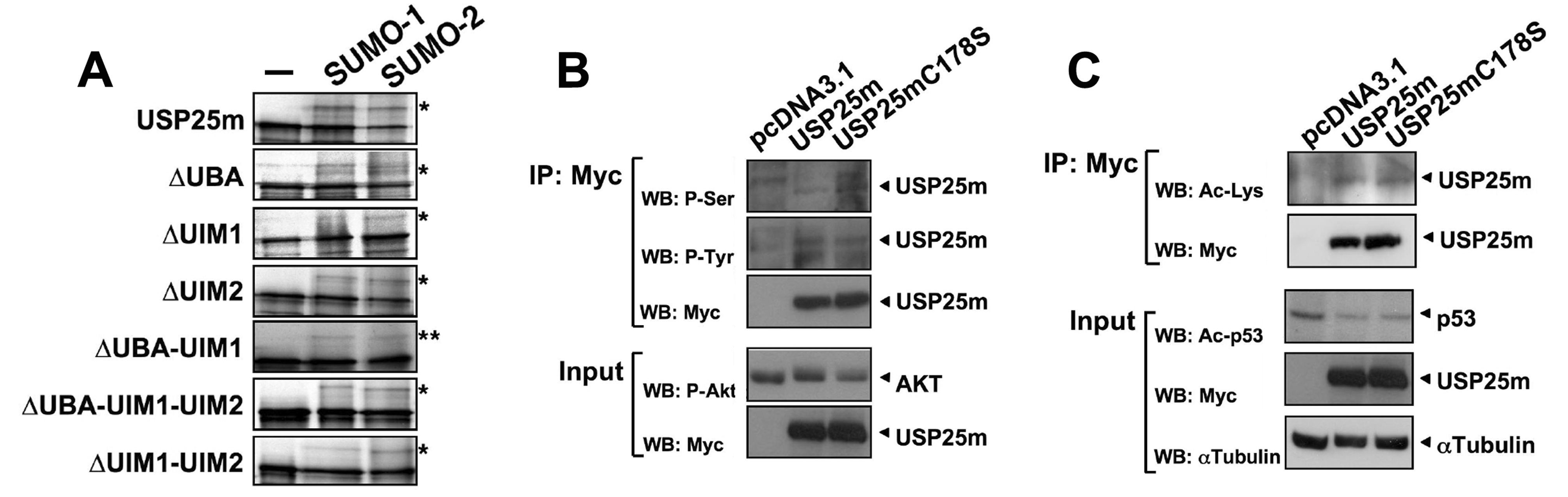


**Material and methods**

*In vitro SUMO-1 and SUMO-2 conjugation assays-*The full length USP25m and deletion constructs were transcribed, translated and ^35^S-Met-labeled in vitro (TNT-T7 Coupled Wheat Germ Extract Systems. Cat. No. L4140 PROMEGA) (2µl) in a 10 µl reaction including an ATP regenerating system (50 mM Tris pH 7.6, 10 mM MgCl2, 2 mM ATP, 10 mM creatine phosphate, 3.5 U/ml of creatine kinase, and 0.6 U/ml of inorganic pyrophosphatase), SUMO-1 or SUMO-2 (1µg), Ubc9 (0,325µg) and purified SAE1/2 (0,08µg) (Biomol Cat. No. UW9330). Reactions were incubated at 30ºC for 2 hours. The reaction products were loaded onto 8.5% SDS-PAGE and analyzed by autoradiography. SUMO-1, SUMO-2 and Ubc9 were kindly provided by Dr. M. Rodriguez.

*Phosphorylation and acetylation assays-*HEK293T cells were plated in 10 cm dishes and transfected the following day with 6 µg of pcDNA-Myc-USP25m or pcDNA-Myc-USP25mC178S with Lipofectamine 2000 (Invitrogen). Forty-eight hours post-transfection cells were washed in PBS, resuspended in 1 ml of binding buffer (20 mM HEPES pH 7.9, 50 mM KCl, 2 mM MgCl_2_, 0.5 mM EDTA, 10% glycerol 2 mM DTT, 10 mM NaF, 20 mM β–glycerophosphate, 0.05% NP-40, and protease inhibitor cocktail) and sonicated. After centrifugation, cell lysates were incubated with 2 µg of anti c-Myc antibody (Santa Cruz Biotechnology) for 4 h at 4ºC with gentle agitation. The protein-antibody complexes were captured by 1 h incubation with protein G-Sepharose beads (GE-Healthcare) at 4ºC on a rotating mixer. The bound proteins were washed three times, eluted from the beads by boiling with protein loading buffer, loaded onto an 8% SDS-PAGE, and analyzed by Western blot analysis, using anti-phosphoSer polyclonal (Sd7, 1:200, Cell Signaling) and anti-phosphoTyr monoclonal (PY20, 1:1000, Cell Signaling) antibodies for USP25m phosphorylation and anti-acetylated Lys (1:1000, Cell Signaling) for USP25m acetylation. Monoclonal antibodies against phosphorylated AKT (1:1000 Cell Signaling) or acetylated p53 (1:1000, Cell Signaling) were used as controls.
